# Influence of Face Masks on the Use of Contact Lenses

**DOI:** 10.3390/ijerph18147407

**Published:** 2021-07-11

**Authors:** Clara Martinez-Perez, Bruno Monteiro, Mafalda Soares, Fatima Portugues, Sonia Matos, Ana Ferreira, Cristina Alvarez-Peregrina, Miguel Ángel Sánchez-Tena

**Affiliations:** 1 ISEC LISBOA—Instituto Superior de Educação e Ciências,1750-179 Lisboa, Portugal; bruno.monteiro@lentesdecontacto.pt (B.M.); mafalda.soares7@hotmail.com (M.S.); fportugues@hotmail.com (F.P.); atelierdoptica@gmail.com (S.M.); ferre.anasofia@gmail.com (A.F.); masancheztena@ucm.es (M.Á.S.-T.); 2 Faculty of Biomedical and Health Science, Universidad Europea de Madrid, 28670 Madrid, Spain; cristina.alvarez@universidadeuropea.es; 3 Department of Optometry and Vision, Faculty of Optics and Optometry, Universidad Complutense de Madrid, 28037 Madrid, Spain

**Keywords:** contact lenses, mask, COVID-19

## Abstract

Background: The COVID-19 epidemic is largely controlled by the use of face masks. The use of a face mask has been indicated as a strong cause of dry eye, although it is not yet described in the literature. This study aims to compare the impact of the use of masks on the visual quality of patients. The symptoms in the human eye intensified during the pandemic versus the symptoms before the pandemic, in a Portuguese population. Methods: A fifteen-question questionnaire was conducted to find out what changes occurred in the use of soft contact lenses during the pandemic in relation to the use of masks. Statistical analysis was performed with SPSS 27.0 software (SPSS Inc., Chicago, IL, USA). Results: The use of contact lenses decreased compared with before the pandemic (*p* < 0.001). The number of hours of wear decreased significantly compared with before the pandemic (*p* < 0.001). The sensation of dry eyes was found to be worse in those using monthly replacement contact lenses (*p* = 0.034), and the need to remove contact lenses was more frequent in women (*p* = 0.026) after using a mask. Conclusions: Mask use increases dry eye symptoms in contact lens wearers, negatively impacting visual quality.

## 1. Introduction

Over a year has passed since the WHO declared the COVID-19 pandemic, and evidence of ocular manifestation in COVID-19 patients is gradually accumulating. As of May 2021, severe acute respiratory syndrome 2 (SARS-CoV-2) has infected 155,665,214 people globally, and it has been responsible for 3,250,648 deaths [1]. However, information on the ocular signs and symptoms caused by SARS-CoV-2 is still lacking. COVID-19 can affect the eyes in several ways. On the one hand, it can directly affect the ocular surface tissue and cause conjunctivitis, which has already been well described since the beginning of the pandemic [2,3]. Both the conjunctiva and the cornea express the ACE2 molecule, which is the target of SARS-CoV-2 [4]. Therefore, another possible manifestation is viral keratitis. Second, an eye infection could also occur due to systemic problems. Patients admitted to intensive care developed chemosis, conjunctival injection, exposure keratopathy, and secondary infectious keratitis due to positive pressure from mechanical ventilation, electrolyte imbalance, and fluid imbalance between body compartments, etc. [5,6]. Third, the treatment used to control COVID-19 infection could have ocular side effects, such as bull’s eye maculopathy secondary to considerably higher doses than are safe, or possible drug-induced uveitis secondary to the use of antiviral medications [7].

To reduce the spread of the virus, the WHO has mandated the use of masks (medical or surgical masks, N-95 respirators, or similar) as a potential tool to address the COVID-19 pandemic since the initial outbreak in China, although recommendations for their use have been modified according to the time and the availability [8,9]. Thus, at the beginning of the pandemic, many experts advised against the use of masks by the general population, since it was thought that the risk of self-contamination was greater than the benefits and also that due to the shortage of masks, the necessary supply for health workers could be depleted. Later, the experts’ way of thinking changed, and they recommended that self-protection was the best form of infection prevention and control [10].

Therefore, today, despite the great pharmacological advances and the development of vaccines, masks and face shields are crucial to protect us from COVID-19. For this reason, more and more ophthalmologists are diagnosing more cases of dry eye as a result of prolonged mask use [11,12]. Thus, scientists at the Centre for Ocular Research and Education (CORE), Waterloo, Canada, termed dry eye after mask use as mask-associated dry eye (MADE) [13,14,15]. This is because the use of masks reduces the spread of air, and the exhaled air moves upwards. In this way, an air current is created over the cornea, leading to faster evaporation of the tear film, which produces dry spots on the ocular surface, eye irritation, and discomfort [16]. In turn, the contact between the warmer exhaled air and the cooler glasses lenses results in condensation and the formation of small water droplets that scatter light, reduce visual acuity, and negatively affect vision [17,18]. It should be noted that in the survey conducted by Matusiak et al. [19], 21.3% of the participants complained of fogging of the glasses, but only 0.3% reported difficulties in wearing the glasses or limited vision. Therefore, MADE can worsen dry eye symptoms in patients who already have dry eye or postmenopausal dry eye; people who use a smartphone, digital device, or computer for more than 2 h; elderly men or postmenopausal women; post-cataract IOL surgery cases; post-Lasik cases; contact lens wearers, who often have a lower-quality corneal tear film; and masked people who work long hours in air-conditioned settings and/or using digital screens [11]. 

This situation is worrying, since the incidence of dry eye worldwide ranges between 5% and 50%, including contact lens wearers and non-wearers. In Southeast Asia, the incidence varies between 20.0% and 52.4% [20,21,22]. An exception to this region is Singapore, where two studies showed a prevalence of only 6.5% [23] and 12.3% [24]. In Spain, the United Kingdom, and the United States, the rate is lower, at 18.4% [25], 14.5% [26], and 20% [27,28], respectively. In France the rate is slightly higher, at 39.2% [29]. Most studies reported a significantly higher prevalence in women compared with men, ranging between 1.33 and 1.74 times higher [21,24,30].

Health professionals who used masks with adhesive tape to prevent the spread of air in the eyes also complained of eye irritation. It has been suggested that adhesive tape adhered to the skin can affect the lower eyelid, causing a possible mechanical ectropion associated with lagophthalmos [13].

To date, there is hardly any scientific evidence on the effect of masks on contact lens wearers. This is why the objective of the present study is to analyze the impact of using face masks on contact lens wearers as well as their subjective symptoms.

## 2. Materials and Methods

### 2.1. Study Design and Data Collection 

For data collection, a questionnaire was distributed online through internal platforms of different optical centers during the month of April 2021 throughout the Lisbon region, in Portugal. 

The survey is comprised of nine questions in Portuguese, which are based on the use and state of ocular comfort with soft contact lenses after the use of the face mask compared with before the pandemic. To create the questionnaire, we relied on various validated dry eye questionnaires (OSDI, DEQS, UNC DEMS, NEI VFQ 25, SPEED, DEQ-5, DEEP, and CLDEQ-8). The elaboration of the questions was carried out through an interactive process according to the characteristics of confinement in Portugal [31].

The research team generated a list of possible questions in order to obtain the following information: demographic information (sex, age, profession, etc.); contact lens type, replacement, and compliance with contact lens care before and after the pandemic; contact lens wear frequency before the pandemic and how their use has changed during and after the pandemic; frequency of mask use; ocular symptoms associated with the use of contact lenses (CL) and the influence of the mask on these symptoms.

To ensure consistency in the responses regarding the frequency of mask use, it was necessary for respondents to specify if they were teleworking and if they did not use the mask during work, or if there was social distancing and the use of a mask in their job. In this way, we ensured that the frequency and hours of contact lens wear would not affect the responses.

In turn, the total hours of contact lens wear were also taken into account, since even if a person did not wear the mask at certain times, lenses could also influence dry eye symptoms.

The questions about the symptoms of dry eye with contact lenses were based on the different questionnaires mentioned above. Special attention was paid to the questionnaire “The Contact Lens Dry Eye Questionnaire-8 (CLDEQ-8)” since it is the only validated questionnaire on eye symptoms in contact lens wearers. Eighteen questions were established, considering the apparent validity and content validity of each item [32]. The questions were reviewed by a team of Portuguese optometrists, all authors of this manuscript (B.M., M.S., F.P., S.M., and A.F.), to refine the final wording of the questions during the translation process. The final questionnaire was composed of the following questions:

1. Frequency of use and hours of wear of contact lenses (CL) before the pandemic (classified as “Daily”, “2–3 days/week”, and “Sporadically”)
2. Current frequency of use and hours of use of CL (classified as “Daily”, “2–3 days/week”, and “Sporadically”)
  2.1 If the time of use has varied, explain the reasons.
3. Frequency of cleaning routine before and after the pandemic (rated as “Improves”, “Worsens”, and “Stays the same”)
4. Symptoms of eye discomfort with the use of contact lenses (rated as “Never”, “Sometimes”, and “Often”) and with the mask (rated as “Improves”, “Worsens”, and “Stays the same”)
5. Foreign body sensation, dryness, irritation, itching, or burning with the use of contact lenses (rated as “Never”, “Sometimes”, and “Often”) and with the mask (rated as “Improves”, “Worsens”, and “Stays the same”).
6. Eye dryness with the use of contact lenses (rated as “Never”, “Sometimes”, and “Often”) and with the mask (rated as “Improves”, “Worsens”, and “Stays the same”)
7. Sensation of blurriness with the use of contact lenses (rated as “Never”, “Sometimes”, and “Often”) and with the mask (rated as “Improves”, “Worsens”, and “Stays the same”).
8. Need to close the eyes due to ocular discomfort with the use of contact lenses (rated as “Never”, “Sometimes”, and “Often”) and with the mask (rated as “Improves”, “Worsens”, and “Stays the same”)
9. Need to remove contact lenses due to eye discomfort (rated as “Never”, “Sometimes”, and “Often”) and with the mask (rated as “Better”, “Worse”, and “Stays the same”)

### 2.2. Statistical Analysis 

The statistical analysis was carried out using the SPSS 27.0 software (SPSS Inc., Chicago, IL, USA). The normal distribution of the variables was conducted with the Kolmogorov–Smirnov test, with a significance level of 0.05. As a result of a non-parametric distribution, the Wilcoxon non-parametric test was used. To verify the association between the categorical variables, the chi-squared test or Fisher’s exact test, as appropriate, was used. To evaluate the statistical significance, a cut-off point of *p* < 0.05 was considered.

## 3. Results

### 3.1. Descriptive Analysis 

**Figure 1:**
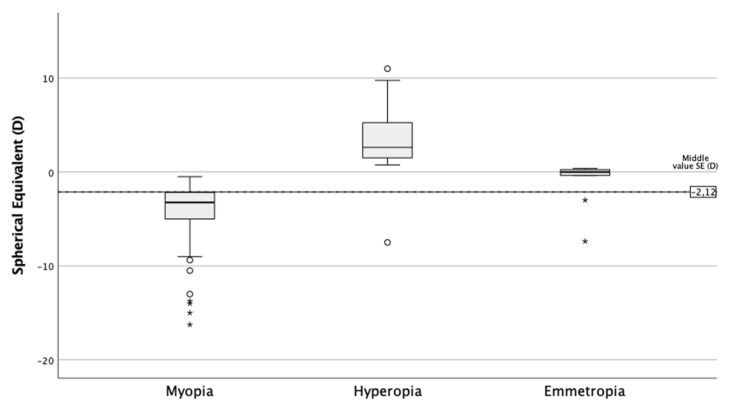
Spherical equivalent as a function of ametropia.

A total of 177 subjects participated, with a mean age of 38.39 ± 10.9 years. Regarding sex, 27.7% were men and 72.3% women. Of all the participants, 35% reported they suffered some type of allergy. Regarding the replacement of CL, 62.7% of the subjects used monthly replacement CL, 28.8% daily replacement, and 8.5% two-week replacement. Most of the participants (71.8%) were myopic (SE ≤ −0.5D), 23.2% were hyperopic (SE ≥ +0.50D), and 5.1% had an SE between –0.50D and +0.50D (Figure 1).

### 3.2. CL Usage Frequency 

The use of CL decreased compared with before the pandemic (*p* ≤ 0.001). Previously, 79.7% of the population used CL daily, 11.9% sporadically, and 8.5% 2–3 times/week, whereas the current usage frequency is 62.7%, 20.3%, and 16.9%, respectively. The main reasons were in 79.6% due to spending more time at home, in 16.3% due to discomfort with the mask, and in the remaining 4% due to fear of infections and for rest from contact lenses. 

**Figure 2:**
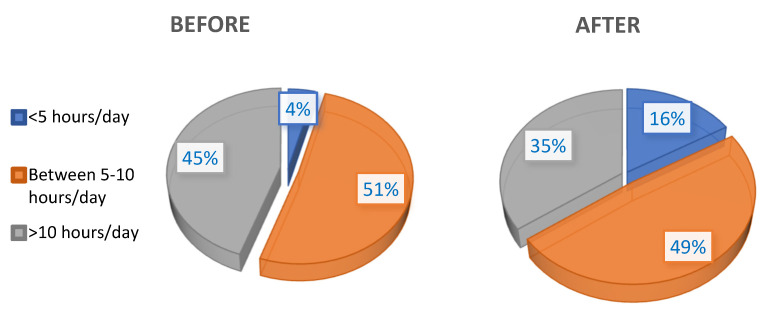
Hours of contact lens wear before and after the pandemic.

In turn, as shown in Figure 2, the number of hours of wear also decreased significantly compared with before the pandemic (*p* ≤ 0.001).

Concerning the cleaning routine, that is, taking care of and disinfecting the lenses, in 92.6% of cases it remained the same, and in 6.8% it improved. 

### 3.3. Eye Symptoms 

**Figure 3:**
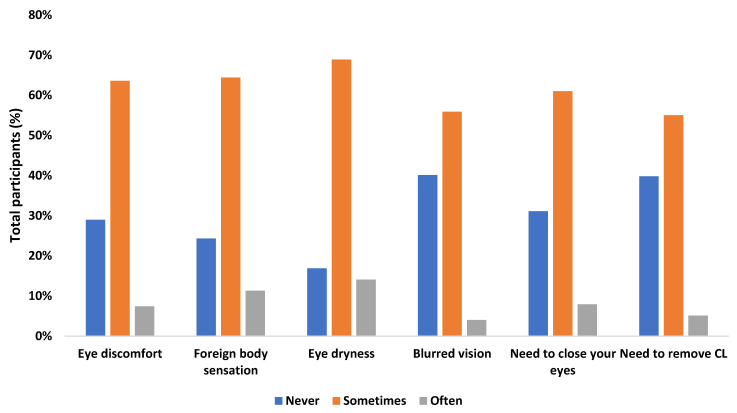
Presence of ocular symptoms in contact lens wearers before the pandemic.

**Figure 4:**
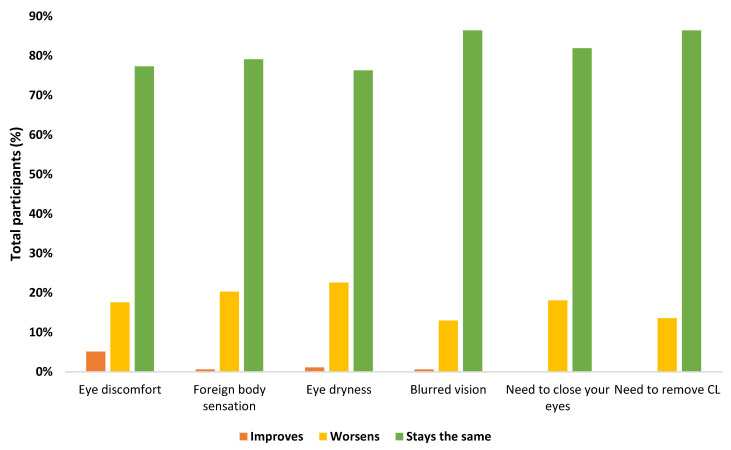
Presence of ocular symptoms in contact lens wearers after the use of face masks.

Among the participants, 61.5% sometimes presented some symptoms associated with dry eye (Figure 3). These symptoms remained constant in 81.2% of participants after using the mask, in 17.5% of participants they worsened, and in 1.2% they improved (Figure 4). In turn, it was found that the sensation of dry eyes was worse in monthly contact lens wearers (*p* = 0.034), and the need to remove contact lenses after mask use was more frequent in women (*p* = 0.026). No association was found between the presence of ocular symptoms and the refractive state (*p* > 0.05), nor with allergies (*p* > 0.05).

## 4. Discussion

This study aimed to analyze the ocular symptoms associated with dry eye in soft contact lens wearers after using the mask as a method of protection against COVID-19. Before the pandemic, the Tear Film & Ocular Surface Society (TFOS) had already reported that discomfort and the presence of discomfort while wearing contact lenses were associated with a reduction in the compatibility between contact lenses and the ocular environment [33]. The causes of discomfort are multiple and can be related to the contact lens itself (material, design, and care) or the environment (compliance and ocular surface conditions). There is still little literature available about the side effects of the mask at the ocular level, and none in contact lens wearers, although a higher incidence of eye irritation has been found in similar cases. For instance, continuous positive airway pressure machines can irritate the ocular surface due to air leakage or regurgitation through the nasolacrimal system [34,35]. Thus, the key factor is the exit of the exhaled air, with a temperature of approximately 36–37 °C, which passes through the upper edge of the mask towards the ocular surface. This hot air current causes instability, increased evaporation, hyperosmolarity, and a decrease in tear film renewal, leading to an increase in dry eye symptoms. In turn, the severity of the symptoms is related to the thickness of the tear lipid layer [19]. In exhaled air, the oxygen level is decreased, and the air presents an increased concentration of carbon dioxide, which leads to a decrease in pH in the tear and a deterioration of the ocular surface, as well as a reduction in the stromal pH, causing a sensation of corneal pain [36,37,38,39]. For the elaboration of the questionnaire, we relied on the twelve dry eye questionnaires validated to date, and to analyze quality of life, five questionnaires were used (OSDI, IDEEL, NEI-VFQ, SANDE, and DEQS), including, among others: the McMonnies Dry Eye History Questionnaire (which includes questions about age, gender, contact lens wear, previous dry eye diagnosis, and triggers, and also assesses the frequency of symptoms of dryness, roughness, pain, redness, tiredness, and medications used); the Women’s Health Study (WHS) questionnaire (which is based on three questions about the previous diagnosis of dry eye, sensation of dryness, and frequency of eye irritation); the Dry Eye questionnaire (which includes questions about the use of contact lenses, age, and gender, and also assesses the frequency and intensity of the following symptoms: comfort, dryness, blurred vision, pain and irritation, grit and itchiness, burning and stinging, foreign body sensation, sensitivity to light, and itching; this questionnaire also includes questions about the time of day it worsens, the effect on activities of daily living, medications, allergies, dry mouth, nose or vagina, treatments, and global assessment of the patient); the North Carolina Dry Eye Management Scale (which includes questions about the severity of eye symptoms and how it affects daily life); Subjective Evaluation of Symptom of Dryness (which assesses how dryness-related ocular discomfort influences the clinical practice of patients); Standard Patient Evaluation of Eye Dryness (which discusses the frequency and severity of the patient’s dry eye symptoms); and Dry Eye Epidemiology Project (which includes questions about the use of eye drops, compresses, drops, frequency of symptoms such as itching, pain, dryness, itching, grit, burning, irritation, tearing, photophobia, red, sticky, or sore, as well as the presence of mouth dry eye, eye allergies, how often you wear contact lenses, and whether you have previously been diagnosed with dry eye) [40,41,42,43,44,45,46].

In this study, it was found that the frequency of contact lens use decreased compared with before the pandemic. Although the main reason for this is that people have been spending more time in their homes, it should not be forgotten that a part of the population is concerned about the increase in infections. This is consistent with studies by Sun et al. [47] and Olivia et al. [48], in which they found that the tear film is an essential barrier against pathogens, but it can be compromised if mask use causes the barrier to evaporate faster. In addition, dry eye symptoms cause people to rub their eyes and face more, thereby spreading the virus. All of these factors lead to increased concern about eye infections secondary to prolonged mask wear. During the COVID-19 pandemic, this risk is especially worrisome, since the virus has already been documented to spread through contact with the eye [49]. Additionally, it has already been shown that after 3 years of wearing CL, between 10% and 50% of users abandon them. Among the general population, 70% of people report symptoms of eye discomfort at the end of the day, the most common being the sensation of dry eye. Approximately 40% of soft lens wearers report this symptom, of which 25% report moderate to severe symptoms, reducing their wear time [50,51,52,53].

In this sense, in our study, it was found that 61.5% of the participants had dry eye symptoms, of which 63.6% were ocular discomfort. In turn, these symptoms remained constant in most of the participants. This incidence agrees with the study by Boccardo et al. [54], in which approximately one third of the participants (32.1%) never experienced dry eye symptoms, 54.3% sometimes, and 13.6% often. Of the 2447 symptoms they reported, only 0.8% of participants felt their symptoms improved when they wore a mask, 72.3% did not notice any change, and 26.9% said their symptoms worsened. In other words, 18.3% of the patients had dry eye associated with the mask, a figure similar to that of our study (17.5%). However, it should be noted that, unlike our results, they did not find significant differences in dry eye symptoms in users who wore glasses as compared to CL users. This difference may be because this study was carried out in the months of September and October 2020, and our data were collected during the month of April 2021, in a period of home confinement, which is why the usage of masks was higher. At the same time, it must be taken into account that the study by Boccardo et al. was carried out in Italy and ours in Portugal, and mask use by the population is different in each country. 

Another study is that of Giannaccare et al. [12], in which the average daily mask use was 6 hours/day, and ocular symptoms worsened in 10.3% of the participants. Likewise, 19.6% of the participants reported the need for an artificial tear. 

On the other hand, we also have to consider that dry eye, together with the sensation of a foreign body, redness, tearing, itching, eye pain, and discharge were the most important ocular manifestations in patients with COVID-19. In the systemic review by Nasiri et al. [55], it was concluded that the mechanism of dry eye or foreign body sensation is unclear in COVID-19 patients and may not be directly associated with SARS-CoV-2. The appearance of dry eye during the COVID-19 epidemic can be related to the use of face masks, mainly when the masks are loose against the face and nose, and the airflow is greater towards the eyes. In turn, decreased access to lubricating agents for fear of contamination from hands and drug containers also deteriorates the manifestations of dry eye [47]. 

For this reason, vision professionals should be aware of this new type of dry eye and educate patients to fit their masks well so that exhaled air does not go directly into the eyes, at the same time as the promotion of the widespread use of masks being maintained. To do this, eye care professionals must ensure that the mask is properly fitted, especially in patients with glasses or contact lenses. Adhesive tape can be carefully attached to the upper edge, but it should not interfere with blinking.

Using artificial tears to lubricate the eyes (following recommended guidelines) can help reduce symptoms. Finally, it should also be advised that people reduce the use of air conditioning and take breaks from the use of digital devices to minimize digital visual fatigue [11].

Future research will report on the incidence and magnitude of dry eye after using masks, as well as the long usage time of digital screens, which may be a contributing factor to an epidemic of dry eye diseases recently. In turn, it is expected that after this first investigation, the influence of the use of contact lenses and masks will be analyzed in more detail since masks will still be used for a long time, mainly indoors, where air conditioning is increasing. It should be considered that to reduce the ocular problems associated with mask use and reduce the rate of abandonment of contact lenses, it is necessary to treat the symptoms associated with dry eye and recommend protective measures for the ocular surface, especially in patients who already have dry eye or other pathologies of the anterior ocular surface.

Concerning the limitations of this study, it must be considered that the evaluation of dry eye exclusively through symptoms may be partial since the clinical signs are not considered. However, the questionnaires allow the obtainment of information on the self-perceived symptoms of the population, especially during the pandemic period, which limits contact between people. 

## 5. Conclusions

This study shows that the use of masks increases eye symptoms in contact lens wearers. In turn, the prolonged use of a mask throughout the day increases the symptoms of discomfort and the sensation of dry eye. This may lead us to predict that since mask use will still last for months or even years, the prevalence of dry eye will increase significantly in the coming years.
